# Objective assessment of motor activity in a clinical sample of adults with attention-deficit/hyperactivity disorder and/or cyclothymic temperament

**DOI:** 10.1186/s12888-022-04242-1

**Published:** 2022-09-14

**Authors:** Vigdis Elin Giaever Syrstad, Kristin Mjeldheim, Wenche Førland, Petter Jakobsen, Rolf Gjestad, Jan Øystein Berle, Kathleen Ries Merikangas, Ketil Joachim Oedegaard, Ole Bernt Fasmer

**Affiliations:** 1 Division of Psychiatry, Haukeland University Hospital, Bergen, Norway; 2 Department of Clinical Medicine, University of Bergen, Bergen, Norway; 3 Division of Psychiatry, NORMENT, Haukeland University Hospital, Bergen, Norway; 4 Madlamarkveien 2a, Hafrsfjord, Norway; 5 Lagårdsveien 91, Stavanger, Norway; 6 Center for Crisis Psychology, Faculty of Psychology, University of Bergen, Bergen, Norway; 7 Genetic Epidemiology Research Branch, Intramural Research Program, National Institute of Mental Health, Bethesda, Maryland USA

**Keywords:** ADHD, Cyclothymic temperament, Motor activity, Actigraphy

## Abstract

**Background:**

Most research on patterns of motor activity has been conducted on adults with mood disorders, but few studies have investigated comorbid attention-deficit/hyperactivity disorder (ADHD) or temperamental factors that may influence the clinical course and symptoms. Cyclothymic temperament (CT) is particularly associated with functional impairment. Clinical features define both disorders, but objective, biological markers for these disorders could give important insights with regard to pathophysiology and classification.

**Methods:**

Seventy-six patients, requiring diagnostic evaluation of ADHD, mood or anxiety disorders were recruited. A comprehensive diagnostic evaluation, including the CT scale of the Temperament Evaluation of Memphis, Pisa, Paris and San Diego – Auto-questionnaire (TEMPS-A), neuropsychological tests and actigraphy, was performed. ADHD was diagnosed according to the DSM-IV criteria. There was a range of different conditions in this clinical sample, but here we report on the presence of CT and ADHD in relation to motor activity. Twenty-nine healthy controls were recruited. We analyzed motor activity time series using linear and nonlinear mathematical methods, with a special focus on active and inactive periods in the actigraphic recordings.

**Results:**

Forty patients fulfilled the criteria for ADHD, with the remainder receiving other psychiatric diagnoses (clinical controls). Forty-two patients fulfilled the criteria for CT. Twenty-two patients fulfilled the criteria for ADHD and CT, 18 patients met the criteria for ADHD without CT, and 15 patients had neither. The ratio duration of active/inactive periods was significantly lower in patients with CT than in patients without CT, in both the total sample, and in the ADHD subsample.

**Conclusions:**

CT is associated with objectively assessed changes in motor activity, implying that the systems regulating motor behavior in these patients are different from both healthy controls and clinical controls without CT. Findings suggest that actigraphy may supplement clinical assessments of CT and ADHD, and may provide an objective marker for CT.

**Supplementary Information:**

The online version contains supplementary material available at 10.1186/s12888-022-04242-1.

## Background

Attention-deficit/hyperactivity disorder (ADHD) and mood disorders are significant health problems, both in children and adults, and are often found in the same patients [1]. There is a growing need to identify biological markers for these disorders to gain insight into their pathophysiology and classification [2]. Alterations of motor activity are prominent features of both conditions [3, 4], but such findings have not often been applied in routine clinical practice.

Mathematical analyses of the variability and complexity of actigraphy showed that patients with ADHD did not differ from normal controls with regard to total activity level, but in short-term recordings had increased variability in the high frequency part of the spectrum using Fourier analysis [3]. When studying ADHD in children, the symptoms of hyperactivity ADHD are primarily an inability to regulate activity in specific situations. Hyperactivity does however not refer to high levels of activity across times and situations. Excessive activity during reading class might be expected, but not in sports class [5]. Patients with depressive disorders differed from controls in short-term recordings, having a lower activity level and increased variability measured by standard deviation [6]. When studying actigraphic registrations from both patients and healthy controls, it is apparent that intraindividual variability is very high, usually approximately equal to the mean values. There are often long periods with uninterrupted zero or very low activity counts, which do not occur only at nighttime, and conversely, long periods with continuous activity [7]. It has previously been thought that the time intervals between behavioral events usually occur randomly, following an exponential distribution and thus giving a straight line on a log-linear plot (probability vs. waiting time), meaning that very long interevent times should be very rare. There is now increasing evidence that many different types of human actions are characterized by bursts of activity separated by long periods of inactivity, such as sending emails or making telephone calls [8, 9]. Such actions will follow a heavy-tail distribution, and on a log-log plot (probability vs. waiting time), this will appear as a straight line, suggestive of a power-law distribution.

With this background, and using actigraphy to measure activity, it has previously been shown that the distribution of active and inactive periods differs between patients with depression and patients with schizophrenia, and that these distributions are different again from those of healthy controls [7, 10–12]. Furthermore, Nakamura et al. found that the organization of motor activity of mice displayed a similar pattern, with the distribution of resting period durations obeying a power-law distribution [13].

In a previous study using data from Conner’s Continuous Performance Test II, patients with ADHD differed from clinical controls on measures of variability and complexity, in a patient sample identical to the present study sample. However, these changes applied only to the subgroup of ADHD patients that fulfilled the criteria for a cyclothymic temperament (CT) [14]. Augmented mood, emotional instability, and hypersensitivity to external stimuli are characteristic of CT [15]. CT might be understood as a trait related to a bipolar spectrum disorder, which is a chronic condition of subthreshold depression and hypomania [16] and not to be confused with the DSM-IV cyclothymic disorder. Cyclothymic disorder however is a subtype of bipolar disorder, and characterized by chronic but less extreme mood states [17, 18]. The prevalence of CT is relatively common in patients with affective disorders, as well as in adult patients with ADHD and is particularly associated with functional impairment [19, 20].

The aim of the present study was to reanalyze our previous actigraphic registrations of patients with ADHD and mood disorders to determine whether the distribution of active and resting periods can be used to differentiate 1) ADHD patients from normal controls and from patients with mood and anxiety disorders; 2) patients with CT from patients without this type of temperament; and 3) ADHD patients with CT from ADHD patients without CT.

## Methods

### Ethics statement

The Norwegian Regional Medical Research Ethics Committee West approved the study protocol. Written informed consent was obtained from all participants involved in the study.

### Subjects

Consecutive new referrals for a diagnostic evaluation of either ADHD, or mood/anxiety disorders at a private psychiatric practice were recruited as participants. Exclusion criteria were inability to speak Norwegian and not being able to comply with the study protocol. One-hundred and four patients were included. We were unable to obtain complete 6-day recordings for 28 patients for several reasons, due to logistics problems, patients forgetting to wear the actigraphic devices and incomplete recordings. Therefore, actigraphic data for 76 patients are presented in the current paper. Most of the patients (*n* = 49) were not taking any psychotropic drugs. The remainder were taking antidepressants or mood stabilizers, and two used a low dosage of antipsychotic. Patients using drugs at referral continued their normal use of these during the actigraphic recordings.

The control group consisted of five medical students, four patients without serious medical or psychiatric symptoms from a primary care office, and twenty employees from either Bergen University or a psychiatric nursing home. None of the subjects had a history of mood or psychotic disorders. The controls were recruited during a separate study, presented in two previous papers [6, 21]. The controls used actigraphy equipment equivalent to that used by the patients.

### Psychiatric assessment

All diagnostic assessments of the patients were performed by either KM or WF using a standard clinical interview, supplemented when possible with information from collateral sources. In addition, the following assessment instruments were employed:

The Mini-International Neuropsychiatric Interview (MINI Plus, version 5.0.0), a module based semi-structured interview for DSM-IV and ICD-10 diagnoses [22, 23]; the Montgomery-Asberg Depression Rating Scale (MADRS), a standard instrument for the assessment of depression [24]; and the Adult ADHD Self-Report Scale (ASRS), the World Health Organization’s rating scale designed to measure current symptoms of ADHD in adults. The ASRS includes 18 questions that follow the DSM-IV criteria for ADHD on a five-point scale (0 = never, 4 = very often), with a possible range of scores from 0–72. Items 1–9 cover symptoms of inattention and items 10–18 cover hyperactivity and impulsivity [25, 26]. We also used the Wender Utah Rating Scale, 25-question version (WURS-25), a 25-item self-rating scale designed to assess symptoms and signs of ADHD in childhood, using a five-point Likert scale (0 = never, 4 = very often), yielding a possible score range of 0–100 [27]. The WURS-25 has been used in previous studies in Norway [25].

The Mood Disorder Questionnaire (MDQ) was used as a screening instrument for bipolar disorder. It is a self-report form that consists of 13 questions scored “Yes” or “No”. Positive answers to at least seven questions and confirmation that the symptoms have occurred together and caused problems is suggestive of a bipolar disorder [25, 28]. We also used the Cyclothymic Temperament Scale, a self-report form that consists of 21 questions covering CT according to Akiskal’s definition. This scale is part of the larger TEMPS-A auto-questionnaire [19, 29, 30]. Finally, we used the Hospital Anxiety and Depression Scale (HADS), a self-assessment form for detecting current states of depression and anxiety, which has been extensively used, including in Norway [31]. The final diagnostic evaluation was made after an assessment of all available information, and a consensus diagnosis, based on DSM-IV criteria, was made after discussion of each case.

### Recording of motor activity

Motor activity was monitored with an actigraphic device (Actiwatch; Cambridge NeuroTech, Cambridge, UK) worn on the right wrist. Activity is measured on the Actiwatch by a piezoelectric accelerometer that is programmed to record the integration of intensity, amount, and duration of movement in all directions. The sampling frequency is 32 Hz and movements over 0.05 g will be recorded. A corresponding voltage is produced and is stored as an activity count in the memory unit of the Actiwatch. In actigraphy studies to record in 32Hz and analyze the one-minute mean is the common method [32]. The actigraph we used (Actiwatch; Cambridge NeuroTech, Cambridge, UK) delivers data in this format. However to use this method and data format, and loose motion data with a frequency of 16-20Hz, is not an important issue as explained by Khusainov [33]. The number of counts is proportional to the intensity of the movement. The right wrist was chosen to make the procedure more convenient for the participants because most of them wore their watches on the left wrist and it was considered cumbersome to have two such devices on the same arm. Previous studies have shown that there are small differences between the right and left wrists [34, 35]. Total activity counts were recorded at one-minute intervals for a period of six days.

### Mathematical analyses

For the motor activity time series we calculated the mean activity, the standard deviation (SD), expressed as percentage of the mean, and the root-mean-square successive differences (RMSSD), expressed as percentage of the mean [3, 36]. Each recorded interval (one minute) was then defined as either active or inactive. An active count was defined as above (or equal to) 10% of the mean for the whole recording period (8640 minutes) for that participant. An inactive count was defined as below 10% of the mean for the whole recording period (8640 minutes) for that participant. We have employed the 10 % threshold value, since this is the same threshold we used in our previous work with depressed and schizophrenic patients [7] and we found meaningful differences between the groups with this value. Nakamura who established this method tested several different threshold values, with very similar results for the different thresholds [11]. An active period was defined as a continuous sequence of counts defined as active. The same principle goes for inactive periods. Each recording was divided into active or inactive periods, with lengths from one minute upwards. For each recording, the cumulative probability (P) that an active or inactive period had a length of ≥A minutes was determined. For both active and inactive periods, the following values of A were used: 1–5 with one-minute intervals, and 6–101 with five-minute intervals. For each participant, the following numbers were calculated: mean activity count for the whole recording period, mean duration for active and inactive periods, and the longest active and inactive periods. In accordance with previous studies using this method [7, 10, 14], we plotted P vs. A on a log-log graph using lengths from 1–35 min for active periods and 1–20 min for inactive periods, and calculated the slope of the line (scaling exponent) that best fitted the data, using the least squares method. The slope was negative, and absolute values are given in the tables. We also calculated the number of active periods (expressed as % of all the active periods) with a length (A) of 36 min or longer, and the number of inactive periods (% of all the inactive periods) with a length (A) of 21 min or longer.

### Statistics

One-way analysis of variance (ANOVA) was employed to evaluate differences between groups, with post hoc Bonferroni tests. Bonferroni corrections adjust for multiple comparisons when testing more than one hypothesis, the significance level adjust to avoid false positive results [37]. Chi-square tests were used to deal with categorical data and Pearson’s correlation coefficient was used to evaluate correlations. Effects of age and sex were evaluated using analysis of covariance (ANCOVA). SPSS software (v. 25; IBM SPSS, Armonk, NY, USA) was used for the statistical analyses.

## Results

The clinical group consisted of 35 women and 41 men with an average age of 37.9 years (SD = 10.9, range = 17–61). The control group consisted of 18 women and 11 males with an average age of 37.8 years (SD = 13.3, range = 21–66). Table 1 shows the clinical characteristics of the patients, grouped according to the presence or absence of ADHD, and the presence or absence of CT. Gender distribution were not significantly different between the groups for neither ADHD nor CT. For the presence or absence of ADHD groups, the scores on WURS and ASRS were significantly higher for the patients with ADHD. For the presence or absence of CT groups, the patients with CT were significantly younger, and scored significantly higher on ASRS, MADRS and MDQ, as well as on the HADS depression and anxiety scales. Furthermore, Table 1 displays the clinical characteristics of the patients grouped into four groups: patients with both ADHD and CT present; patients with ADHD only; patients with CT only; and those with neither ADHD nor CT. There were one-way ANOVA statistically significant differences between the groups for WURS, ASRS, MADRS, MDQ and HADS Depression scores. With Bonferroni corrections we found significantly higher scores on the WURS and ASRS for the patients with ADHD and CT present compared to both patient groups without ADHD. In addition, the MDQ scores were significantly higher for the patients with ADHD and CT compared to both patients groups without CT.

**Table 1:** Characteristics of the clinical sample according to the presence of ADHD and CT

	**ADHD**	**Not ADHD**	P ^*^	**CT**	**Not CT**	P ^*^	**ADHD + CT**	**ADHD Not CT**	**Not ADHD + CT**	**Not ADHD Not CT**	ANOVA ^**^
	(*n* = 39) ^a^	(*n* = 37) ^a^		(*n* = 41) ^b^	(*n* = 33) ^b^		(*n* = 22) ^c^	(*n* = 18) ^c^	(*n* = 19) ^c^	(*n* = 15) ^c^	
**Age**	38.4 ± 10.6	37.3 ± 11.4	0.675	35.8 ± 10.2	40.8 ± 11.1	**0.049**	38.2 ± 11.2	39.1 ± 10.2	33.0 ± 8.7	42.8 ± 12.1	F(70,3) = 2.55, *p* = 0.063
**Gender (m/f)**	23/16	18/19	0.373	23/19	16/15	0.793	12/10	11/7	10/9	6/9	
**WURS**	50.2 ± 20.4	30.8 ± 15.5	**< 0.001**	43.1 ± 20.2	38.0 ± 21.2	0.301	51.8 ± 19.3	46.4 ± 22.8	33.0 ± 16.6^**#**^	28.5 ± 14.8^**#**^	F(67,3) = 6.11, ***p* = 0.001**
**ASRS**	47.8 ± 13.4	32.7 ± 13.4	**< 0.001**	45.2 ± 12.9	35.9 ± 15.8	**0.008**	50.6 ± 11.5	43.5 ± 14.9	38.5 ± 11.5^**#**^	27.2 ± 12.2^**#**^	F(66, 3) = 10.55, ***p* < 0.001**
**HADS**											
** Depression**	4.6 ± 3.8	5.5 ± 4.2	0.369	6.3 ± 4.0	3.8 ± 3.7	**0.011**	5.6 ± 3.9	3.5 ± 3.3	7.2 ± 4.1	4.2 ± 4.3	F(65,3) = 2.91, ***p* = 0.041**
** Anxiety**	9.6 ± 4.6	9.0 ± 4.9	0.658	10.6 ± 4.4	8.1 ± 4.8	**0.027**	10.7 ± 4.6	8.2 ± 4.4	10.4 ± 4.3	7.9 ± 5.4	F(65,3) = 1.68, *p* = 0.181
** MADRS**	14.0 ± 7.7	13.8 ± 8.2	0.929	16.6 ± 8.5	11.0 ± 6.4	**0.003**	16.7 ± 7.9	10.7 ± 5.7	16.4 ± 9.3	11.4 ± 7.3	F(67,3) = 3.03, ***p* = 0.035**
** MDQ**	6.4 ± 3.9	6.4 ± 3.8	0.986	8.4 ± 3.4	3.9 ± 2.7	**< 0.001**	8.8 ± 3.5	3.7 ± 2.3^**#**^**^**	7.9 ± 3.3	4.2 ± 3.1^**#**^	F(65,3) = 11.79, ***p* < 0.001**
** CT**	11.5 ± 4.7	11.3 ± 4.2	0.114	14.4 ± 2.5	7.1 ± 2.6	**< 0.001**	14.8 ± 2.6	7.1 ± 2.8^**#**^**^**	13.9 ± 2.4	7.0 ± 2.5^**#**^**^**	F(68,3) = 47.50, ***p* < 0.001**

All data are given as mean (standard derivation), if not otherwise specified

^a^ Total number of ADHD classified patients = 76. Number of subjects varies between the different measures as only 74 subjects delivered a valid CT scale score; *n* = 36–38 for ADHD and *n* = 29–30 for non-ADHD

^b^ Total number of CT rated patients = 74. Number of subjects varies between the different measures; *n* = 39–40 for CT and *n* = 31–32 for non-CT

^c^ Total number of CT rated patients = 74. Number of subjects varies between the different measures; *n* = 20–22 for ADHD + CT, *n* = 16–17 for ADHD without CT, *n* = 17–18 for non-ADHD with CT, and *n* = 14-15 for the non-ADHD non-CT group

^*^ Independent samples t-test with Levene’s test for equality of variance, significance level *p* < 0.05

^**^ One-way ANOVA, significance level *p* < 0.05

Post hoc Bonferroni tests (significance level *p* < 0.05):

^#^ *p* < 0.05 ADHD + CT vs ADHD Not CT or Not ADHD + CT or Not ADHD Not CT

^^^ *p* < 0.05 Not ADHD + CT vs ADHD Not CT or Not ADHD Not CT

**Fig. 1:**
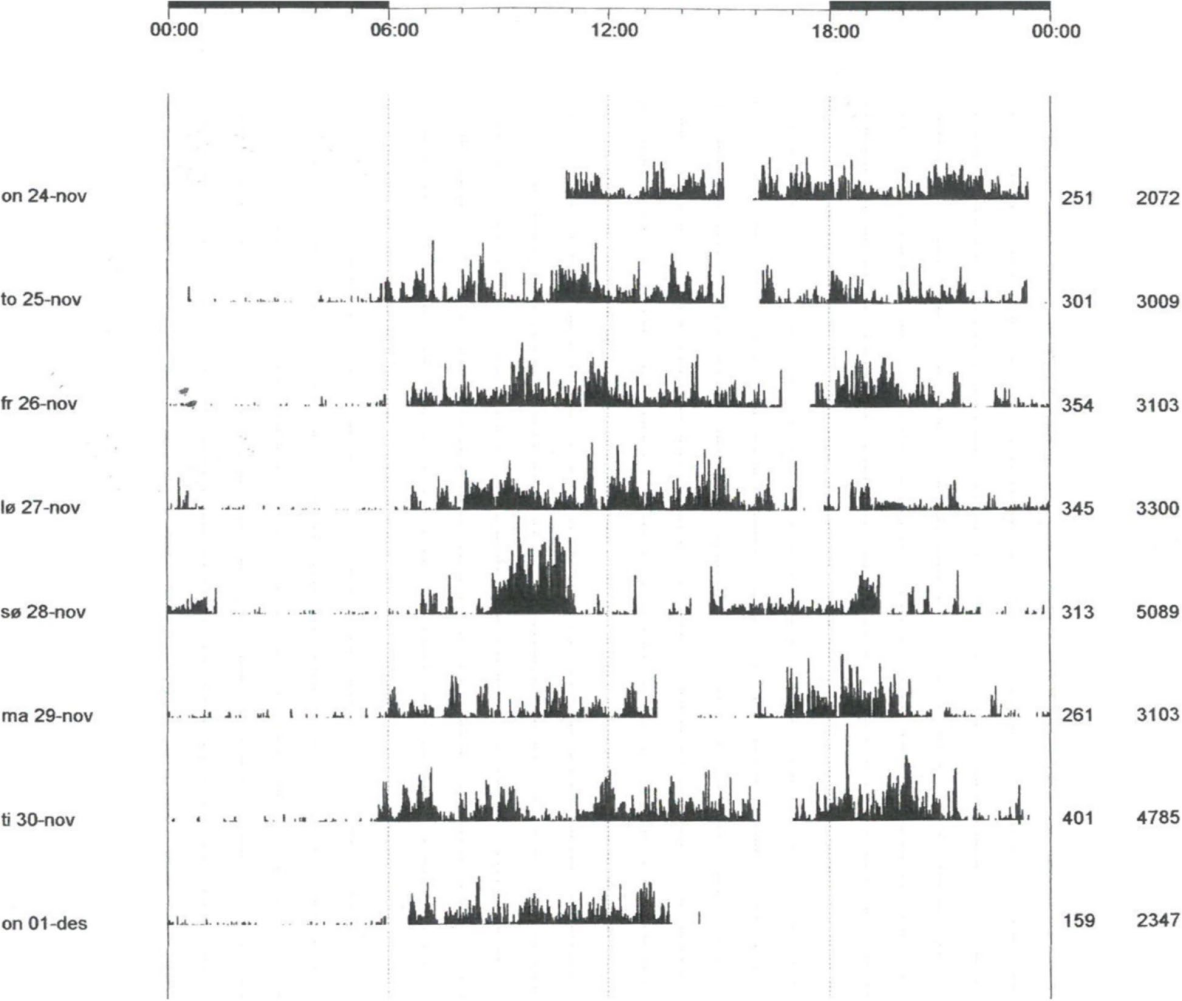
An actigraphic registration over six days from a patient with cyclothymic temperament and ADHD

Figure 1 illustrates a six-day Actigraphic recording from a patient with both ADHD and CT. Table 2 presents the features of the actigraph recordings from the patients with and without ADHD and the controls. Both patient groups present Bonferroni significant lower mean activity scores than the controls. The RMSSD variability measure and the longest inactive sequence estimate were statistically significant elevated (ANOVA) for both patient groups compared to controls, although these differences was only Bonferroni significant for the ADHD group. Furthermore, the longest inactive sequence and the scaling exponent for active periods were statistically significant higher (ANOVA) and active sequences ≥ 36 minutes were significant lower for both patients group.

**Table 2:** Actigraphic recordings, six days motor activity; ADHD, without ADHD and controls

	**Controls**	**ADHD**	**Not ADHD**	ANOVA ^*^
	(*n* = 29)	(*n* = 39)	(*n* = 37)	
Activity count/min	286 ± 87	231 ± 90 ^#^	221± 94 ^#^	F(102,2) = 4.724, ***p* = 0.011**
SD (% of mean)	147 ± 20	159 ± 22	157 ± 26	F(100,2) = 2.427, *p* = 0.093
RMSSD (% of mean)	103 ± 16	116 ± 19 ^#^	113 ± 21	F(100,2) = 4.654, ***p* = 0.012**
Active period duration	9.6 ± 2.0	7.8 ± 2.3	8.0 ± 2.7	F(102,2) = 2.933, *p* = 0.058
Inactive period duration	5.9 ± 1.0	6.0 ± 1.6	6.0 ± 1.5	F(102,2) = 0.047, *p* = 0.954
Active/inactive duration	1.55 ± 0.31	1.33 ± 0.38	1.40 ± 0.68	F(102,2) = 1.677, *p* = 0.192
Longest active sequence	254 ± 166	187 ± 73	218 ± 159	F(102,2) = 2.013, *p* = 0.139
Longest inactive sequence	77 ± 19	192 ± 204 ^#^	178 ± 208	F(102,2) = 4.051, ***p* = 0.020**
Active sequences ≥36 min	6.5 ± 2.2	5.2 ± 2.5	5.1 ± 2.3	F(102,2) = 3.589, ***p* = 0.031**
Inactive sequences ≥21 min	7.3 ± 2.3	6.2 ± 2.2	6.4 ± 2.	F(102,2) = 2.216, *p* = 0.114
Scaling exponent:				
Active periods	0.733 ± 0.125	0.810 ± 0.145	0.807 ± 0.126	F(102,2) = 3.359, ***p* = 0.039**
Inactive periods	0.825 ± 0.101	0.890 ± 0.128	0.896 ± 0.142	F(102,2) = 3.053, *p* = 0.052

All data are given as mean ± standard derivation. Durations are given in minutes. The numbers for active sequences 36 minutes and the number of inactive sequences 21 minutes are given in percent of the total number of sequences

^*^ One-way ANOVA, significance level *p* < 0.05

Post hoc Bonferroni tests (significance level *p* < 0.05):

^#^ *p* < 0.05 Controls vs. ADHD or not ADHD

When controlling for age using analysis of covariance (ANCOVA), we found a significantly association for diagnostic group on the longest inactive sequence measure, besides significant interactions between age and diagnostic group for the inactive period duration and inactive sequences ≥21 minutes variables (Supplemental table 1). When controlling for gender we found significantly associations for diagnostic groups for inactive period duration, ratio of active/inactive duration and inactive sequences ≥21 min. Furthermore, there were significant interactions between gender and diagnostic group for SD and RMSSD (Supplemental table 2).

Table 3 presents the features of the actigraph recordings when grouping the patients according to the presence of CT. Seven measures were statistically significant (ANOVA) when comparing the patients with CT, the patients without CT and controls, and the differences were in addition Bonferroni significant between CT patients and controls. For both patient groups the estimates of mean activity, active period duration, active/inactive duration and active sequences ≥36 minutes were lower than for controls, and the estimates of RMSSD, the longest inactive sequence and the scaling exponent for active periods were higher.

**Table 3:** Actigraphic recordings, six days motor activity; CT, without CT and controls

	**Controls**	**CT**	**Not CT**	ANOVA ^*^
	(*n* = 29)	(*n* = 41)	(*n* = 33)	
Activity count/min	286 ± 87	221 ± 82 ^#^	232 ± 104	F(100,2) = 4.684, ***p* = 0.011**
SD (% of mean)	147 ± 20	159 ± 22	158 ± 26	F(100,2) = 2.519, *p* = 0.086
RMSSD (% of mean)	103 ± 16	116 ± 21 ^#^	113 ± 19	F(100,2) = 4.654, ***p* = 0.012**
Active period duration	9.6 ± 2.0	7.5 ± 2.5 ^#^	8.4 ± 2.4	F(100,2) = 4.262, ***p* = 0.017**
Inactive period duration	5.9 ± 1.0	6.2 ± 1.7	6.0 ± 1.3	F(100,2) = 0.338, *p* = 0.714
Active/inactive duration	1.55 ± 0.31	1.24 ± 0.33 ^#^	1.48 ± 0.68	F(100,2) = 4.288, ***p* = 0.016**
Longest active sequence	254 ± 166	193 ± 86	214 ± 160	F(100,2) = 1.670, *p* = 0.193
Longest inactive sequence	77 ± 19	211 ± 241 ^#^	160 ± 151	F(100,2) = 4.969, ***p* = 0.009**
Active sequences ≥36 min	6.5 ± 2.2	4.9 ± 2.5 ^#^	5.5 ± 2.3	F(100,2) = 4.200, ***p* = 0.018**
Inactive sequences ≥21 min	7.3 ± 2.3	6.2 ± 2.5	6.5 ± 2.3	F(100,2) = 2.079, *p* = 0.130
Scaling exponent				
Active periods	0.733 ± 0.125	0.832 ± 0.142 ^#^	0.778 ± 0.123	F(100,2) = 4.858, ***p* = 0.010**
Inactive periods	0.825 ± 0.101	0.894 ± 0.129	0.881 ± 0.136	F(100,2) = 2.794, *p* = 0.066

All data are given as mean ± standard derivation. Durations are given in minutes. The numbers for active sequences 36 minutes and the number of inactive sequences 21 minutes are given in percent of the total number of sequences

^*^ One-way ANOVA, significance level *p* < 0.05

Post hoc Bonferroni tests (significance level *p* < 0.05):

^#^ *p* < 0.05 Controls vs. CT

When controlling for age using analysis of covariance (ANCOVA), we found no significant associations for CT group for any of the parameters, but there was a significant interactions between age and diagnostic group for the longest inactive sequence (Supplemental table 3). When controlling for gender we found a significantly association for diagnostic group on the inactive period duration, ratio of active/inactive duration, inactive sequences ≥21 min and scaling exponent for inactive periods, there were no significant interactions between gender and diagnostic group (Supplemental table 4).

**Table 4:** Actigraphic recordings, six days motor activity; ADHD and CT, ADHD without CT, and controls

	**Controls**	**ADHD + CT**	**ADHD Not CT**	ANOVA ^*^
	(*n* = 29)	(*n* = 22)	(*n* = 18)	
Activity count/min	286 ± 87	205 ± 78 ^#^	259 ± 95	F(66,2) = 5.556, ***p* = 0.006**
SD (% of mean)	147 ± 20	163 ± 21 ^#^	155 ± 22	F(66,2) = 3.701, ***p* = 0.030**
RMSSD (% of mean)	103 ± 16	122 ± 21 ^#^	111 ± 16	F(66,2) = 7.453, ***p* = 0.001**
Active period duration	9.1 ± 1.9	7.1 ± 2.0 ^#^	8.5 ± 2.5	F(66,2) = 5.557, ***p* = 0.006**
Inactive period duration	5.9 ± 1.0	6.3 ± 1.9	5.7 ± 1.0	F(66,2) = 1.225, *p* = 0.300
Active/inactive duration	1.55 ± 0.31	1.17 ± 0.28 ^#^	1.51 ± 0.40 ^^^	F(66,2) = 9.434, ***p* < 0.001**
Longest active sequence	254 ± 166	186 ± 86	183 ± 58	F(66,2) = 2.722, *p* = 0.073
Longest inactive sequence	77 ± 19	228 ± 235 ^#^	142 ± 147	F(66,2) = 6.148, ***p* = 0.004**
Active sequences ≥36 min	6.5 ± 2.2	4.6 ± 2.5 ^#^	5.8 ± 2,4	F(66,2) = 4,231, ***p* = 0.019**
Inactive sequences ≥21 min	7.3 ± 2.3	6.2 ± 2.5	6.2 ± 1.9	F(66,2) = 2.297, *p* = 0.109
Scaling exponent				
Active periods	0.733 ± 0.125	0.847 ± 0.144 ^#^	0.765 ± 0.132	F(66,2) = 4.660, ***p* = 0.013**
Inactive periods	0.825 ± 0.101	0.892 ± 1.36	0.891 ± 0.119	F(66,2) = 2.700, *p* = 0.075

All data are given as mean ± standard derivation. Durations are given in minutes. The numbers for active sequences 36 minutes and the number of inactive sequences 21 minutes are given in percent of the total number of sequences

^*^ One-way ANOVA, significance level *p* < 0.05

Post hoc Bonferroni tests (significance level *p* < 0.05):

^#^ *p* < 0.05 Controls vs. ADHD + CT

^^^ *p* < 0.05 ADHD + CT vs. ADHD not CT

When grouping the ADHD patients into those with CT and those without CT, and then comparing with healthy controls applying ANOVA and post hoc Bonferroni corrections, the only measure significantly differed between the ADHD groups was the duration of active periods/duration of inactive period’s ratio. Furthermore, the ADHD patients with CT was significantly different from controls on this ratio, as well as for seven other variables presented in Table 4. The same comparisons in the group of patients without ADHD (19 with CT and 15 without CT) found none of the measures to be significantly different (the ratio duration of active periods/duration of inactive duration was 1.32 ± 0.36 vs. 1.44 ± 0.93).

When cross classifying the sample by ADHD and CT, and comparing with healthy controls applying ANOVA and post hoc Bonferroni corrections, we found ANOVA significant differences between all five groups for mean activity, RMSSD, duration of the longest inactive sequence, and scaling exponent-active periods. For the mean activity variable, we found Bonferroni significant differences between the control group and the patients with ADHD and CT and the patients without both ADHD and CT. Furthermore, we found significant differences between patients with ADHD and CT compared to controls for RMSSD, active period duration, longest inactive sequence, active sequences ≥ 36 minutes and scaling exponent-active periods (Table 5).

**Table 5:** Actigraphic recordings, six days motor activity; all patients groups and controls

	**Controls**	**ADHD + CT**	**ADHD Not CT**	**Not ADHD + CT**	**Not ADHD Not CT**	ANOVA ^*^
	(*n* = 29)	(*n* = 22)	(*n* = 18)	(*n* = 19)	(*n* = 15)	
Activity count/min	286 ± 87	205 ± 78 ^**#**^	259 ± 95	239 ± 85	200 ± 110 ^**#**^	F(98,4) = 3.666, ***p* = 0.008**
SD (% of mean)	147 ± 20	163 ± 21	155 ± 22	154 ± 22	162 ± 31	F(98,4) = 1.868, *p* = 0.122
RMSSD (% of mean)	103 ± 16	122 ± 21 ^**#**^	111 ± 16	111 ± 20	115 ± 24	F(98,4) = 3.344, ***p* = 0.013**
Active period duration	9.1 ± 1.9	7.1 ± 2.0 ^**#**^	8.5 ± 2.5	7.9 ± 3.0	8.3 ± 2.5	F(98,4) = 2.373, *p* = 0.057
Inactive period duration	5.9 ± 1.0	6.3 ± 2.0	5.7 ± 1.0	6.0 ± 1.5	6.3 ± 1.6	F(98,4) = 0.740, *p* = 0.567
Active/inactive duration	1.55 ± 0.31	1.17 ± 0.28	1.51 ± 0.40	1.32 ± 0.37	1.44 ± 0.93	F(98,4) = 2.463, *p* = 0.050
Longest active sequence	254 ± 166	186 ± 86	183 ± 58	202 ± 88	252 ± 227	F(98,4) = 1.386, *p* = 0.244
Longest inactive sequence	77 ± 19	228 ± 235 ^**#**^	142 ± 147	191 ± 254	181 ± 159	F(98,4) = 3.668, ***p* = 0.037**
Active sequences ≥36 min	6.5 ± 2.2	4.6 ± 2.5 ^**#**^	5.8 ± 2.4	5.2 ± 2.6	5.2 ± 2.2	F(98,4) = 2.363, *p* = 0.058
Inactive sequences ≥21 min	7.3 ± 2.3	6.2 ± 2.5	6.2 ± 1.9	6.2 ± 2.5	7.0 ± 2.7	F(98,4) = 1.256, *p* = 0.292
Scaling exponent						
Active periods	0.733 ± 0.125	0.847 ± 0.144^**#**^	0.765 ± 0.132	0.814 ± 0.142	0.793 ± 112	F(98,4) = 2.653, ***p* = 0.038**
Inactive periods	0.825 ± 0.101	0.892 ± 136	0.891 ± 119	0.896 ± 125	0.869 ± 0.157	F(98,4) = 1.439, *p* = 0.227

All data are given as mean ± standard derivation. Durations are given in minutes. The numbers for active sequences 36 minutes and the number of inactive sequences 21 minutes are given in percent of the total number of sequences

^*^ One-way ANOVA, significance level *p* < 0.05

Post hoc Bonferroni tests (significance level *p* < 0.05):

^#^ *p* < 0.05 Controls vs. ADHD + CT or Not ADHD Not CT

## Discussion

The main finding of the present study is that the distribution of active and inactive periods in actigraphically assessed motor activity differentiates patients with CT from normal controls and from patients without CT. This adds to previously published findings of actigraphy in patients with depression and schizophrenia. These results demonstrate that the construct of affective temperament, in this case CT, is associated with objectively assessed changes in motor activity, implying that the systems regulating motor behavior in these patients are different from both clinical controls and normal controls.

The ratio of active to inactive period duration seems to be the most interesting parameter in this study. When looking at the whole patient group, this ratio was significantly lower in patients with CT than those without CT, and furthermore, in the ADHD group, this ratio was also lower in patients with CT than patients without CT. We have previously studied the same group of patients using the CPT-II test [14] and found that changes in variability and complexity in the ADHD patients were only seen in the subgroup that also fulfilled the criteria for CT. The present results therefore support the notion that dividing ADHD patients into subgroups may in many ways be more meaningful if mood symptoms are used rather than the traditional division into hyperactive, inattentive and combined types [27, 38, 39]. There might be a risk of bias that patients with CT also had higher scores on MADRS and HADS depression scale.

In our previous study of depressed and schizophrenic patients [7] we found that the cumulative probability distribution (P) of active periods and duration (A) ≤35 min followed a straight line on a log-log plot, suggestive of a power-law distribution. Similarly, for inactive periods, with duration ≤20 min, P vs. A also followed a straight line on a log-log plot. In the present study, the results were very similar; using log-log plots of P vs. A for active periods ≤35 min and inactive periods ≤20 min, all curves followed fairly straight lines. We did not perform a rigorous study to see if these distributions follow a strict power-law function [36]; our purpose was to determine this, but to use these relationships to look for differences between diagnostic groups. Nakamura et al. [11] found that for both healthy controls and depressed patients the distribution of active periods followed a stretched exponential function, while inactive periods followed a power-law function. Chapman et al. [9] found that for patients with bipolar disorder, active period data were best described by a power-law function while inactive period duration obeyed a truncated power-law function. However, differences in the methods (placement of sensors), selection of patients and length of time sequences make a direct comparison between the three studies difficult [9].

When calculating the slope of these lines (the scaling exponent) we found only small differences between patients with and patients without ADHD. However, when using the presence or absence of CT to group the patients we found a significantly higher absolute value of the scaling exponent for active periods in the CT patients. It is not easy to translate these numbers into meaningful clinical concepts. However, after considering the measures we used—average length of active periods and the percentage of active periods with a length ≥36 min—this becomes easier to grasp. The percentage of active periods with a very long duration (≥36 min) was also lower in CT patients, and patients without CT had intermediate values. From a clinical perspective, these findings are compatible with the notion inherent in the definition of the CT construct that these patients are more variable in terms of behavior than other patients, shifting more rapidly from activity to inactivity, and are less likely to engage in long activity sequences. In addition, patients with CT had a lower total activity level compared with normal controls, and the average duration of their active periods was significantly shorter, while patients without CT had intermediate values. With regard to inactive periods, scaling exponent, duration and percentage of very long periods did not differ significantly between the three groups. These findings are consistent with the results from our study of depressed patients. Similar to the CT group in the present study, the depressed patients were characterized by lower duration of active periods and a lower percentage of very long active periods [7].

The findings from the variability measures (SD and RMSSD) are also compatible with the notion of higher variability in the CT group; both measures were higher in the CT patients, although only RMSSD significantly so.

Patients with ADHD did not differ significantly from those without ADHD in either age or gender distribution. ADHD is increasingly recognized as a disorder that manifests in adulthood where, in contrast to ADHD that manifests in childhood, the prevalence of ADHD in females is approximately equal to that of males. The mean age of patients with CT was somewhat lower than for patients without CT, which is consistent with a large population study of MDQ that showed a decreasing prevalence of bipolarity with increasing age [40].

Actigraphy has been useful in sleep studies, and it is possible that analysis of active and inactive periods, combined with mathematical analyses of periods with continuous motor activity, will yield a biological “signature” that can be used for diagnostic purposes. In behavioral studies, it is obviously important that objective registrations of motor behavior are added to subjective clinical impressions. It will also be interesting to see if a combination of such measures, with the help of machine-learning techniques, can be employed to predict treatment effects. Other possibilities include using new technological developments, such as smartphones combined with activity registrations [9].

We used six days of registration in the present study, and 12 days in our previous study. However, we have also shown that these methods can be applied to analyses of shorter periods (24 hours) [10], and this is, of course, more applicable to routine clinical practice.

There are some limitations to this study, as this is a small sample which may have impacted the statistical power. We have thus not controlled for sex, age and body mass index. However, to our knowledge no differences in activation have previously been identified between sexes, but physical properties such as increased age and body mass index have previously been found to affect mean motor activity [2]. Treatment with psychotropic medication may, of course, have also influenced results, but the majority of patients in our sample were not taking psychotropic medication.

Sleep parameters, which are also altered in many of our patients, were not analyzed separately in this study. Although this may have influenced our results, it would be difficult to separate such effects from other effects on rest and activity rhythms. All the patients in this study were outpatients, but we did not collect information that may have allowed us to compare activity schedules with those of the controls. This is a source of bias that is difficult to evaluate. Diagnoses were assessed non blind, but the actigraphic registrations and subsequent mathematical analyses did not require subjective evaluations. Participants were asked to remove the actigraphic devices while taking a bath or showering, but this accounts for only short time periods, and is unlikely to have biased the results. Despite the declared limitations, our main findings remain robust and well-grounded.

## Conclusions

We found that the distribution of active and inactive periods in actigraphically assessed motor activity differentiated patients with CT from normal controls and from ADHD patients without CT. This provides an objective way to differentiate between patients with more severe disorders [34] and an opportunity to tailor treatment accordingly, to avoid setting off rapid cycling and irritable depression [41, 42]. There is substantial support for mood symptoms being a part of adult ADHD [27, 38, 43]. Dividing ADHD into subgroups according to mood symptoms seems to be more meaningful than the traditional division into hyperactive, inattentive and combined types. Moreover, mood instability should be assessed in patients with ADHD, as recommended by Wender’s use of the WURS [27, 43] and the use of the Wender-Reimherr Adult Attention Deficit Disorder Scale (WRAADS) later by Reimherr [39]

CT is associated with objectively assessed changes in motor activity, implying that the systems regulating motor behavior in these patients are different from both from clinical controls without CT and from normal controls. Actigraphy provides a more objective measure for activity and instability and clearly differentiates between patients with CT and not, especially in ADHD patients.

## Supplementary Information

**Additional file 1:** **Supplemental  Table 1.** Effect of age using analysis of covariance ANCOVA.

**Additional file 2:** **Supplemental  Table 2.** Effect of gender using analysis of covariance ANCOVA.

**Additional file 3:** **Supplemental  Table 3.** Effect of age using analysis of covariance ANCOVA.

**Additional file 4:** **Supplemental  Table 4.** Effect of gender using analysis of covariance ANCOVA.

**Additional file 5:** **Supplemental  Figure 1.** Log-log plots cumulativeprobability (P) vs. duration active periods (£35 min) for patients with CT.

**Additional file 6:** **Supplemental  Figure 2.** Log-log plots cumulativeprobability (P) vs. duration inactive peri­ods (£20 min) for patients with CT.

**Additional file 7:** **Supplemental Figure 3.** Active periods for controls. Log-log plots of cumulative probability (P) vs. duration of activeperiods (£35 min) for controls. The straight line represents the lin­earregression line, using the least squares method.

**Additional file 8:** **Supplemental Figure 4.** Active periods for patients without ADHD. Log-log plots of cumulative probability (P) vs. duration of activeperiods (£35 min) for patients without ADHD. The straight line represents thelin­ear regression line, using the least squares method.

**Additional file 9:** **Supplemental Figure 5.** Inactiveperiods for controls. Log-log plots of cumulativeprobability (P) vs. duration of inactive periods (£20 min)for controls. The straight line represents the lin­ear regression line, usingthe least squares method.

**Additional file 10:** **Supplemental Figure 6.** Inactiveperiods for patients without ADHD. Log-log plots ofcumulative probability (P) vs. duration of inactive periods (£20 min)for patients without ADHD. The straight line represents the lin­ear regressionline, using the least squares method.

## Data Availability

Raw data might be available upon request by contacting the corresponding author; however, the request must comply with confidentiality and ethics rules of the Ethics Committee of our institution.

## Abbreviations

A: Length of active or inactive period in minutes
ADHD: Attention-deficit/hyperactivity disorder
ANCOVA: Analysis of covariance
ANOVA: Analysis of variance
ASRS: Adult ADHD Self-Report Scale
CT: Cyclothymic temperament
DSM-IV: Diagnostic and Statistical Manual of Mental Disorders, 4^th^ edition
HADS: Hospital Anxiety and Depression Scale
ICD-10: International Classification of Diseases and Related Health Problems, 10^th^ revision
MADRS: Montgomery Asberg Depression Rating Scale
MDQ: Mood Disorder Questionnaire
MINIPlus: Mini-International Neuropsychiatric Interview
RMSSD: Root mean square successive differences
SD: Standard deviation
SPSS: Statistical Package for the Social Sciences
TEMPS-A: Temperament Evaluation of the Memphis, Pisa, Paris, & San Diego Auto-questionnaire
WRAADS: The Wender-Reimherr Adult Attention Deficit Disorder Scale
WURS-25: Wender Utah Rating Scale, 25-question version
