# Supplementary material for: Objective assessment of motor activity in a clinical sample of adults with attention-deficit/hyperactivity disorder and/or cyclothymic temperament
Source: BMC Psychiatry. 2022 Sep 14;22:609. doi: 10.1186/s12888-022-04242-1 (PMC9476590; doi:10.1186/s12888-022-04242-1)
Supplement: Supplementary file 1 — Additional file 1: Supplemental Table 1. Effect of age using analysis of covariance ANCOVA. [file 12888_2022_4242_MOESM1_ESM.docx]

**Supplemental table 1 – Effect of age using analysis of covariance ANCOVA.**

The whole sample (controls, ADHD, not ADHD)

N = 105

Activity count/min F = 2.039 p = 0.156

SD (% of mean) F = 1.406 p = 0.239

RMSSD (% of mean) F = 0.441 p = 0.508

Active period duration F = 0.616 p = 0.434

Inactive period duration*

Active/inactive duration F = 0.047 p = 0.829

Longest active sequence F = 0.213 p = 0.645

Longest inactive sequence F = 5.449 **p = 0.022**

Active sequences ≥36 min F = 0.193 p = 0.661

Inactive sequences ≥21 min*

Scaling exponent

Active periods F = 0.059 p = 0.808

Inactive periods F = 1.388 p = 0.241

*For inactive period duration there is a significant interaction between age and diagnosis (F = 7.131, p = 0.031), and correspondingly for inactive sequences ≥21 min (F = 3.955, p = 0.022), violating the assumption of homogeneity of regression slopes, consequently the effect of gender cannot be calculated for these measures.

**p < 0.05**
