# Supplementary material for: Objective assessment of motor activity in a clinical sample of adults with attention-deficit/hyperactivity disorder and/or cyclothymic temperament
Source: BMC Psychiatry. 2022 Sep 14;22:609. doi: 10.1186/s12888-022-04242-1 (PMC9476590; doi:10.1186/s12888-022-04242-1)
Supplement: Supplementary file 2 — Additional file 2: Supplemental Table 2. Effect of gender using analysis of covariance ANCOVA. [file 12888_2022_4242_MOESM2_ESM.docx]

**Supplemental table 2 – Effect of gender using analysis of covariance ANCOVA.**

The whole sample (controls, ADHD, not ADHD)

N = 105

Activity count/min F = 0.512 p = 0.476

SD (% of mean)*

RMSSD (% of mean)*

Active period duration F = 0.250 p = 0.618

Inactive period duration F = 6.925 **p = 0.010**

Active/inactive duration F = 5.058 **p = 0.027**

Longest active sequence F = 0.096 p = 0.757

Longest inactive sequence F = 3.605 p = 0.060

Active sequences ≥36 min F = 0.002 p = 0.968

Inactive sequences ≥21 min F = 5.294 **p = 0.023**

Scaling exponent

Active periods F = 0.005 p = 0.941

Inactive periods F = 5.382 **p = 0.022**

*For SD there is a significant interaction between gender and diagnosis (F = 3.463, p = 0.035), and likewise for RMSSD (F = 4.276, p = 0.017), violating the assumption of homogeneity of regression slopes, consequently the effect of gender cannot be calculated for these measures.

**p < 0.05**
