# Supplementary material for: Objective assessment of motor activity in a clinical sample of adults with attention-deficit/hyperactivity disorder and/or cyclothymic temperament
Source: BMC Psychiatry. 2022 Sep 14;22:609. doi: 10.1186/s12888-022-04242-1 (PMC9476590; doi:10.1186/s12888-022-04242-1)
Supplement: Supplementary file 3 — Additional file 3: Supplemental Table 3. Effect of age using analysis of covariance ANCOVA. [file 12888_2022_4242_MOESM3_ESM.docx]

**Supplemental table 3 – Effect of age using analysis of covariance ANCOVA.**

The whole sample (controls, CT, not CT)

N = 103

Activity count/min F = 2.127 P = 0.148

SD (% of mean) F = 2.107 P = 0.150

RMSSD (% of mean) F = 0.433 P = 0.512

Active period duration F = 0.554 P = 0.459

Inactive period duration F = 0.002 P = 0.966

Active/inactive duration F = 0.101 P = 0.751

Longest active sequence F = 0.286 P = 0.594

Longest inactive sequence*

Active sequences ≥36 min F = 0.195 P = 0.659

Inactive sequences ≥21 min F = 3.713 P = 0.057

Scaling exponent

Active periods F = 0.008 P = 0.928

Inactive periods F = 0.692 P = 0.407

*For the longest inactive sequence there is a significant interaction between age and diagnosis (F = 3.826, p = 0.025), violating the assumption of homogeneity of regression slopes, consequently the effect of gender cannot be calculated for this measure.
