# Supplementary material for: Objective assessment of motor activity in a clinical sample of adults with attention-deficit/hyperactivity disorder and/or cyclothymic temperament
Source: BMC Psychiatry. 2022 Sep 14;22:609. doi: 10.1186/s12888-022-04242-1 (PMC9476590; doi:10.1186/s12888-022-04242-1)
Supplement: Supplementary file 4 — Additional file 4: Supplemental Table 4. Effect of gender using analysis of covariance ANCOVA. [file 12888_2022_4242_MOESM4_ESM.docx]

**Supplemental table 4 – Effect of gender using analysis of covariance ANCOVA.**

The whole sample (controls, CT, not CT)

N = 103

Activity count/min F = 0.378 P = 0.540

SD (% of mean) F = 0.518 P = 0.474

RMSSD (% of mean) F = 0.126 P = 0.724

Active period duration F = 0.541 P = 0.464

Inactive period duration F = 7.751 **P = 0.006**

Active/inactive duration F = 7.868 **P = 0.006**

Longest active sequence F = 0.124 P = 0.726

Longest inactive sequence F = 3.645 P = 0.059

Active sequences ≥36 min F = 0.026 P = 0.871

Inactive sequences ≥21 min F = 5.636 **P = 0.020**

Scaling exponent

Active periods F = 0.012 P = 0.913

Inactive periods F = 6.633 **P = 0.011**

**p < 0.05**
