# Supplementary material for: Objective assessment of motor activity in a clinical sample of adults with attention-deficit/hyperactivity disorder and/or cyclothymic temperament
Source: BMC Psychiatry. 2022 Sep 14;22:609. doi: 10.1186/s12888-022-04242-1 (PMC9476590; doi:10.1186/s12888-022-04242-1)
Supplement: Supplementary file 5 — Additional file 5: Supplemental Figure 1. Log-log plots cumulativeprobability (P) vs. duration active periods (£35 min) for patients with CT. [file 12888_2022_4242_MOESM5_ESM.docx]

**Supplemental figure 1** Log-log plots cumulative probability (P) vs. duration active periods (≤35 min) for patients with CT.
