# Supplementary material for: Objective assessment of motor activity in a clinical sample of adults with attention-deficit/hyperactivity disorder and/or cyclothymic temperament
Source: BMC Psychiatry. 2022 Sep 14;22:609. doi: 10.1186/s12888-022-04242-1 (PMC9476590; doi:10.1186/s12888-022-04242-1)
Supplement: Supplementary file 8 — Additional file 8: Supplemental Figure 4. Active periods for patients without ADHD. Log-log plots of cumulative probability (P) vs. duration of activeperiods (£35 min) for patients without ADHD. The straight line represents thelin­ear regression line, using the least squares method. [file 12888_2022_4242_MOESM8_ESM.docx]

**Supplemental figure 4 Active periods for patients without ADHD**

**Supplemental figure 4 legend** Log-log plots of cumulative probability (P) vs. duration of active periods (≤35 min) for patients without ADHD. The straight line represents the lin­ear regression line, using the least squares method.
