# Supplementary material for: Objective assessment of motor activity in a clinical sample of adults with attention-deficit/hyperactivity disorder and/or cyclothymic temperament
Source: BMC Psychiatry. 2022 Sep 14;22:609. doi: 10.1186/s12888-022-04242-1 (PMC9476590; doi:10.1186/s12888-022-04242-1)
Supplement: Supplementary file 9 — Additional file 9: Supplemental Figure 5. Inactiveperiods for controls. Log-log plots of cumulativeprobability (P) vs. duration of inactive periods (£20 min)for controls. The straight line represents the lin­ear regression line, usingthe least squares method. [file 12888_2022_4242_MOESM9_ESM.docx]

**Supplemental figure 5 Inactive periods for controls**

**Supplemental figure 5 legend** Log-log plots of cumulative probability (P) vs. duration of inactive periods (≤20 min) for controls. The straight line represents the lin­ear regression line, using the least squares method.
