# Supplementary material for: Objective assessment of motor activity in a clinical sample of adults with attention-deficit/hyperactivity disorder and/or cyclothymic temperament
Source: BMC Psychiatry. 2022 Sep 14;22:609. doi: 10.1186/s12888-022-04242-1 (PMC9476590; doi:10.1186/s12888-022-04242-1)
Supplement: Supplementary file 10 — Additional file 10: Supplemental Figure 6. Inactiveperiods for patients without ADHD. Log-log plots ofcumulative probability (P) vs. duration of inactive periods (£20 min)for patients without ADHD. The straight line represents the lin­ear regressionline, using the least squares method. [file 12888_2022_4242_MOESM10_ESM.docx]

**Supplemental figure 6 Inactive periods for patients without ADHD**

**Supplemental figure 6 legend** Log-log plots of cumulative probability (P) vs. duration of inactive periods (≤20 min) for patients without ADHD. The straight line represents the lin­ear regression line, using the least squares method.
